# GPR54 (KISS1R) Transactivates EGFR to Promote Breast Cancer Cell Invasiveness

**DOI:** 10.1371/journal.pone.0021599

**Published:** 2011-06-28

**Authors:** Mateusz Zajac, Jeffrey Law, Dragana Donna Cvetkovic, Macarena Pampillo, Lindsay McColl, Cynthia Pape, Gianni M. Di Guglielmo, Lynne M. Postovit, Andy V. Babwah, Moshmi Bhattacharya

**Affiliations:** 1 Department of Physiology and Pharmacology, The University of Western Ontario, London, Ontario, Canada; 2 The Children's Health Research Institute, The University of Western Ontario, London, Ontario, Canada; 3 Lawson Health Research Institute, The University of Western Ontario, London, Ontario, Canada; 4 Department of Obstetrics and Gynaecology, The University of Western Ontario, London, Ontario, Canada; 5 Department of Anatomy and Cell Biology, The University of Western Ontario, London, Ontario, Canada; Roswell Park Cancer Institute, United States of America

## Abstract

Kisspeptins (Kp), peptide products of the Kisspeptin-1 (*KISS1*) gene are endogenous ligands for a G protein-coupled receptor 54 (GPR54). Previous findings have shown that *KISS1* acts as a metastasis suppressor in numerous cancers in humans. However, recent studies have demonstrated that an increase in *KISS1* and GPR54 expression in human breast tumors correlates with higher tumor grade and metastatic potential. At present, whether or not Kp signaling promotes breast cancer cell invasiveness, required for metastasis and the underlying mechanisms, is unknown. We have found that kisspeptin-10 (Kp-10), the most potent Kp, stimulates the invasion of human breast cancer MDA-MB-231 and Hs578T cells using Matrigel-coated Transwell chamber assays and induces the formation of invasive stellate structures in three-dimensional invasion assays. Furthermore, Kp-10 stimulated an increase in matrix metalloprotease (MMP)-9 activity. We also found that Kp-10 induced the transactivation of epidermal growth factor receptor (EGFR). Knockdown of the GPCR scaffolding protein, β-arrestin 2, inhibited Kp-10-induced EGFR transactivation as well as Kp-10 induced invasion of breast cancer cells *via* modulation of MMP-9 secretion and activity. Finally, we found that the two receptors associate with each other under basal conditions, and FRET analysis revealed that GPR54 interacts directly with EGFR. The stability of the receptor complex formation was increased upon treatment of cells by Kp-10. Taken together, our findings suggest a novel mechanism by which Kp signaling *via* GPR54 stimulates breast cancer cell invasiveness.

## Introduction

Kisspeptins (Kp) are peptide products of the metastasis-suppressor *KISS1* gene and are the endogenous ligands for G protein-coupled receptor 54 (GPR54), a G_q/11_-coupled receptor [1], [2], [3]. Activation of GPR54 results in the activation of phospholipase C, protein kinase C (PKC), and the mitogen-activated protein kinase cascade (MAPK) [4]. The *KISS1* gene codes for a hydrophobic 145 amino acid sequence, which is subsequently cleaved into a 54 amino acid sequence, which in turn may be cleaved by furin or prohormone convertases (deduced by the presence of pairs of basic residues flanking this sequence) into even shorter biologically active peptides (10, 13, 14 amino acids long), collectively referred to as Kp. The shortest and most active *KISS1* gene product is Kisspeptin-10 (Kp-10), a 10-residue peptide [3]. In numerous cancers such as melanoma, pancreatic cancer, and gastric carcinoma, KISS1/GPR54 signaling is anti-metastatic [5], [6]. GPR54 activity has been shown to repress matrix metalloprotease-9 (MMP-9) activity, increase production of tissue inhibitor of matrix metalloprotease-1 (TIMP-1), inhibit cell migration, and increase activity of focal adhesion kinase (FAK), leading to formation of excessive focal adhesions and stress fibres [3], [4]. Additionally, activation of GPR54 by Kp has been shown to inhibit cell motility, proliferation, invasion, chemotaxis and metastasis [2], [3], [7], [8], [9].

The role of GPR54 in breast cancer has been difficult to discern. Recent studies have shown that the expression of *KISS1* and *GPR54* correlates with breast tumor progression and poor patient prognosis [10]. Indeed, patients who died of breast cancer had the highest expression of *KISS1* mRNA [11]. Furthermore, *GPR54* and *KISS1* mRNA expression is significantly elevated in tumors compared to normal mammary tissue [10]. The high *KISS1* and *GPR54* mRNA levels positively correlated with shorter relapse-free survival [10]. However, whether or not Kp actually promote breast cancer invasiveness and the underlying mechanisms that may be involved are unknown.

The epidermal growth factor receptor (EGFR), a member of the ErbB family of receptor tyrosine kinases, is an important therapeutic target for several epithelial tumors, including breast cancer. EGFR is overexpressed in human breast tumors and the EGFR signaling pathway is implicated in the control of cell survival, proliferation, angiogenesis and metastasis [12]. Upon stimulation, EGFR is known to autophosphorylate tyrosine residues on the C-terminal domain of its dimerized binding partner. These residues then serve as docking sites for a number of proteins, including Grb2/Sos, c-cbl, Src, PI3K (Phosphatidylinositol 3-kinases) and phospholipase Cγ (PLCγ), leading to activation of the MAPK pathway and Akt/protein kinase B. The internalization of EGFR has further been shown to be required for mitogenic signaling *via* the activation of MAPKs, resulting in transcriptional changes leading to increased migration, invasion, and metastasis [13], [14].

EGFR can be transactivated by GPCRs in a variety of cell types including tumor cells [15], [16], [17], [18]. Defined as the phosphorylation of ERK1/2 in response to an unrelated agonist, transactivation appears to require EGFR kinase activity, the proteolytic release of EGFR ligands, and a cascade of secondary messengers, including a rise in cytosolic Ca^2+^ concentration, activation of PKC, and tyrosine kinases such as Src and PYK2 [19], [20]. Recently, it has been shown that the GPCR scaffolding protein β-arrestin 2 promotes EGFR transactivation *via* interactions with Src in vascular smooth muscle cells [19].

The aim of this study was to investigate for the first time whether or not Kp directly stimulate breast cancer cell invasiveness and the underlying mechanism(s) involved. The results obtained establish a functional role for Kp signaling in promoting breast cancer cell motility and invasion, two critical processes required for metastasis, *via* cross-talk with EGFR.

## Methods

### Materials and DNA Constructs

Human Kp-10 was purchased from Phoenix Pharmaceuticals (Burlingame, CA). The following were purchased from Sigma Aldrich (St. Louis, MO): human, recombinant epidermal growth factor (EGF), Protein G-sepharose beads, anti-FLAG mouse and rabbit antibodies. Rabbit polyclonal phospho-Src family Tyr416 antibody was obtained from Cell Signaling Technology, MA. Antibody against Kp-10 was a generous gift from Dr. Alain Caraty (Laboratoire de Physiologie de la Reproduction, France) [21]. FLAG-tagged GPR54 and pEGFP-N1-EGFR were obtained from Dr. Andy Babwah.

### Cell Culture

Cell lines were purchased from ATCC (Manassas, VA) and cultured at 37°C with 5% CO_2_. Human invasive breast carcinoma MDA-MB-231, Hs578T, T47D and C8161 cells were cultured in RPMI 1640 (Invitrogen, Burlington, Ontario, Canada) supplemented with 10% (v/v) fetal bovine serum (FBS, Sigma). Human embryonic kidney (HEK)-293 cells were cultured in Eagle's minimum essential medium (MEM; Invitrogen, Burlington, Ontario, Canada), supplemented with 10% (v/v) FBS (Sigma) and 50 µg/mL gentamicin. HEK cells were transiently transfected with FLAG-GPR54 and EGFR-GFP using a modified calcium phosphate method [22]. Fresh medium was added to the cells 18 h after transfections and the cells were allowed to recover for 8 h before being reseeded onto either coverslips for receptor internalization assays, 35 mm glass-bottomed culture plates for confocal microscopy, or 100 mm culture plates for co-immunoprecipitation experiments. Expression of receptors was determined in each experiment using immunofluorescence.

### Stable Transfections and Gene Knockdowns

MDA-MB-231 cells (5×10^6^) were transfected with FLAG-GPR54 constructs (MDA-MB-231 FLAG-GPR54) (25 µg) by electroporation (250 V, 950 µF) using the Gene Pulser Xcell (Bio-Rad, Mississauga, Ontario, Canada) according to the manufacturer's instructions. A heterogeneous population of stable transfectants was selected by using media containing 750 µg/mL G418 (Invitrogen) and expression verified by immunostaining or Western blot for FLAG-GPR54. Gene knockdown of β-arrestin 2 in MDA-MB-231 cells was achieved using shRNA construct (OriGene Technologies, sequence 5′-CAGAATCTTCCATGCTCCGTCACACTGCA-3′, designated shRNA 1) as we have previously described [23]. A heterogeneous population of stable transfectants was obtained *via* puromycin (1 µg/mL) selection. Knockdown of β-arrestin 2 was verified by Western blot analysis.

### Cell Migration and Invasion Assays

Transwell filters (8 µm pores) were placed into a 24-well plate containing either serum-free media or media supplemented with 10% FBS. For cell invasion assays, the tops of filters were coated with 50 µL of diluted phenol-red free Matrigel (9.4 mg/mL stock, BD Biosciences) in serum-free RPMI 1640. Cells were serum-starved for 4 h. MDA-MB-231 (4.0×10^4^), C8161 (4.0×10^4^) or T47D (1.5×10^5^) cells were plated in the upper chamber in either serum-free media or serum-free media supplemented with 10 nM or 100 nM Kp-10 and incubated for 20 h. For studies involving EGFR inhibitor AG1478 (Calbiochem, EMD Biosciences Inc., La Jolla, CA), cells were pre-treated with AG1478 (500 nM) for 30 min as previously described, and seeded in serum-free RPMI with or without Kp-10 (10 nM) in addition to AG1478 (500 nM) or vehicle control (DMSO) [24]. Cells were then fixed with a 20% acetone∶80% methanol solution and nuclei stained with 0.1% Hoechst 33258 (Invitrogen) and counted on the bottom of the filters. Two replicates were conducted for each condition, and 10 random fields in each replicate were chosen and counted using an Olympus IX-71 inverted microscope. Results are presented as a ratio of cells that migrated relative to cells that migrated or invaded in control conditions (cells seeded in serum-free media and migrating towards 10% (v/v) FBS in RPMI). Results are from at least three independent experiments.

### Three-Dimensional Invasion Assays

For three-dimensional cell invasion assays, cells were seeded in a 1∶1 dilution of phenol red-free Matrigel and culture medium at 2.5×10^6^ cells/mL on Matrigel-coated 35 mm glass-bottomed culture dishes (Mattek, Ashland, MA). Cultures were overlaid with culture medium and grown for up to 4 days in the presence or absence of 10 nM Kp-10. Cell colonies were scored blindly as being either stellate or spheroidal after growth in Matrigel. A colony was deemed stellate if one or more projections from the central sphere of cells were observed. Overlaying medium was supplemented with 750 µg/mL of G418 for MDA-MB-231 cells stably transfected with FLAG-GPR54. Images were taken on an IX-81 microscope (Olympus, Center Valley, PA) using InVivo Analyzer Suite (Media Cybernetics).

### MTT Cell Viability Assays

MTT reduction assays (Roche Applied Science) were conducted according to the protocol of the manufacturer, as previously described [23]. Briefly, cells were incubated with 0.5 mg/mL of MTT labeling reagent for 4 h and subsequently solubilized for 12 to 16 h. Absorbance of the supernatant was read at 575 nm using a Wallac Victor3 V plate reader (Perkin-Elmer) and a reference reading taken at 750 nm.

### Zymographic Analysis of Secreted Metalloproteases

Zymographic analysis was performed as previously described [25]. MBA-MD-231 cells were serum starved (24 h). Cells to be treated with inhibitor and ligand were pre-treated with 500 nM AG1478 (Calbiochem) for 30 minutes prior to stimulation. Cells were then stimulated for 24 h with respective reagents. Following stimulation, conditioned media were collected on ice and briefly centrifuged to remove any cellular debris. Supernatant was concentrated in Ultracel-10K Amicon centrifugal filter unit (Millipore). Samples were collected and concentrated conditioned media was resolved in an SDS/polyacrylamide gel containing 1 mg/mL gelatin. The gels were then washed in Novex Zymogram Developing Buffer (Invitrogen) for 30 min to remove the SDS, and incubated overnight at 37°C to allow for enzymatic digestion of the gelatin. For control experiments, gels were incubated in 1× Novex developing buffer containing 5 mM EDTA for 24 h at 37°C. After incubation, gels were stained using Coomassie blue, destained, and visualized using the VersaDoc imaging system (Bio-Rad).

### Scratch Assays for Cell Motility

MDA-MB-231 cells were seeded into a 12-well dish in RPMI 1640 supplemented with 10% FBS and allowed to grow to confluency. Cells were then serum-starved in RPMI for 4 h and scratched with sterile pipette tips followed by treatment with 10 nM or 100 nM Kp-10 in RPMI supplemented with 10% FBS. Cells were allowed to migrate into the scratch for 12 h and visualized every hour using an IX-81 microscope (Olympus). Distance travelled (in µm) was then measured over the course of the 12 h, using duplicates for each condition and five fields per duplicate. Data was analyzed using ImagePro software (Media Cybernetics) and graphed versus time.

### EGFR Immunoprecipitation

MDA-MB-231 or Hs578T cells were serum-starved for 24 h and then stimulated with various ligands at the indicated concentrations for the indicated times. Cell fractions were solubilized in lysis buffer (20 mM HEPES, 150 mM NaCl, 1.5 mM MgCl_2_, 1 mM EDTA, 2 mM Na_3_VO_4_, 1 mM NaF, 10% glycerol, 1% Triton X-100, protease inhibitors). Lysates (850 µg of total protein) were used for immunoprecipitation studies. EGFR was immunoprecipitated using a polyclonal anti-EGFR antibody (1∶100, Upstate Millipore) and protein G-sepharose beads (Sigma) over-night at 4°C. Immunoprecipitated proteins were separated by SDS-PAGE and phosphorylation of EGFR was examined using a mouse monoclonal anti-phosphotyrosine antibody (PY-20, 1∶1000, Santa Cruz) and visualized by enhanced chemiluminescence. Western blots were then reprobed with rabbit polyclonal anti-EGFR antibody (1∶5000, Upstate Millipore) to assess total EGFR. EGFR expression from cell lysates (50 µg of total protein) was examined using a rabbit polyclonal EGFR antibody. To determine the mechanism of EGFR transactivation, cells were grown to 80% confluency and serum-starved for 24 h, prior to stimulation with 10 nM Kp-10 for the indicated time points. EGFR was immunoprecipitated as described above.

### Src Phosphorylation

MDA-MB-231 β-arrestin 2 knockdown stable cell line and scrambled controls were serum-starved for 24 h and then stimulated with 10 nM Kp-10 for the indicated time intervals. Cell fractions were solubilized in lysis buffer (20 mM HEPES, 150 mM NaCl, 1.5 mM MgCl_2_, 1 mM EDTA, 2 mM Na_3_VO_4_, 1 mM NaF, 10% glycerol, 1% Triton X-100, protease inhibitors). Lysates (100 µg of total protein) were separated by SDS-PAGE and phosphorylation of Src was examined using a rabbit polyclonal anti-phosphotyrosine antibody (phospho-Src family Tyr416, 1∶1000, Cell Signaling Technology) and visualized by enhanced chemiluminescence.

### Confocal Microscopy

MDA-MB-231 FLAG-GPR54 (stable cells) were transfected with EGFR-EGFP and were serum-starved for 4 h. Cells were plated on cover-slips and FLAG-GPR54 receptors were labelled with a monoclonal anti-FLAG antibody (1∶500, Sigma) for 45 min at 4°C, warmed to 37°C and treated with Kp-10 or EGF (as indicated in the figure legends). For all experiments, cells were subsequently fixed and permeabilized with 4% paraformaldehyde followed by 0.2% Triton X-100 and FLAG receptors were labelled by anti-mouse AlexaFluor-546 (1∶1200, Invitrogen). Nuclei were stained with 0.1% Hoechst 33258 (Invitrogen). To detect Kp-10 presence in MDA-MB-231 cells, cells were stained using and anti-rabbit polyclonal Kp-10 antibody (1∶750), followed by labelling by anti-rabbit AlexaFluor-555 (1∶1200, Invitrogen). Cells were visualized using a Zeiss LSM-510 META laser scanning microscope (Zeiss, Oberkochen, Germany).

### Co-immunoprecipitation and Immunoblot

HEK 293 cells were transfected with empty FLAG vector (pFLAG-A1) and EGFR-EGFP or FLAG-GPR54 and EGFR-EGFP using modified calcium phosphate method [22]. Forty eight hours following transfection, cells were serum-starved for 4 h and then stimulated with Kp-10 (10 or 100 nM) and EGF (100 ng/mL) for the indicated times. Cell fractions were solubilized in lysis buffer (20 mM HEPES, 150 mM NaCl, 1.5 mM MgCl_2_, 1% Triton X-100, protease inhibitors). Cell lysates (500 µg of total protein) were used for co-immunoprecipitation studies. FLAG-GPR54 was immunoprecipitated using a mouse monoclonal anti-FLAG antibody and protein G-Sepharose beads (Sigma) over-night at 4°C. Immunoprecipitated proteins were separated by SDS-PAGE and EGFR-GFP expression examined using a mouse monoclonal anti-GFP antibody (1∶2000, Upstate Millipore) and visualized by enhanced chemiluminescence, following the manufacturer's protocol.

### Fluorescence Resonance Energy Transfer (FRET)

FRET was performed using the sensitized emission method on an Olympus FV1000 confocal microscope according to the one photon method described by Elangovan *et al.* and the sensitized emission FRET method outlined in the FV10 Olympus software [26]. HEK 293 cells transiently transfected with GPR54-ECFP and EGFR-EGFP seeded on collagen-coated 35 mm glass-bottom culture dishes (Mattek Corp Ashland, MA) were serum starved for 4 h and then imaged live at 37°C with ×60 Plan Apochromat 1.42 oil objective lens and 3× zoom. Seven images were acquired of all three specimens (single-labeled donor, single-labeled acceptor and double-labeled specimen) under the exact same conditions (3× zoom, 1024×1024 pixels, same laser power, optics, gain, integration time). The single-labeled acceptor specimen was excited by donor and acceptor wavelengths and both single-scan images were acquired in the acceptor channel. The single-labeled donor specimen was scanned at the donor excitation wavelength and the image was acquired twice, once in the donor channel and the second time in the acceptor channel. The double-labeled specimen was scanned at the acceptor wavelength and a single scan image was acquired in the acceptor channel; next it was excited by the donor wavelength and images were acquired in the acceptor and donor channels. Using the Precision FRET (PFRET) algorithm donor and acceptor spectral-bleed through were removed, variations in fluorophore expression levels were corrected, the energy transfer efficiency (E%) was calculated, and the distance between donor and acceptor molecules using the same set of cells used for FRET imaging [26]. The Förster distance for the ECFP-EGFP pair is 4.7–4.9 nm. To quantify our results, five regions of interest (ROIs) of the same exact size were placed at different areas of the plasma membrane in each image, and at approximately the same locations in the subsequent time points in each experiment. The mean PFRET efficiency for each area was then determined. The means ± SEM are shown for values obtained for the number of independent experiments indicated in the figure legends.

### Statistical Analysis

One-way analysis of variance (ANOVA) with a Dunnett's post-hoc test or Student's t-test was performed using GraphPad Prism 4 (GraphPad Software, Inc.). Differences with P<0.05 were considered statistically significant.

## Results

### Kp-10 promotes migration and invasion of breast cancer cells

**Figure 1:**
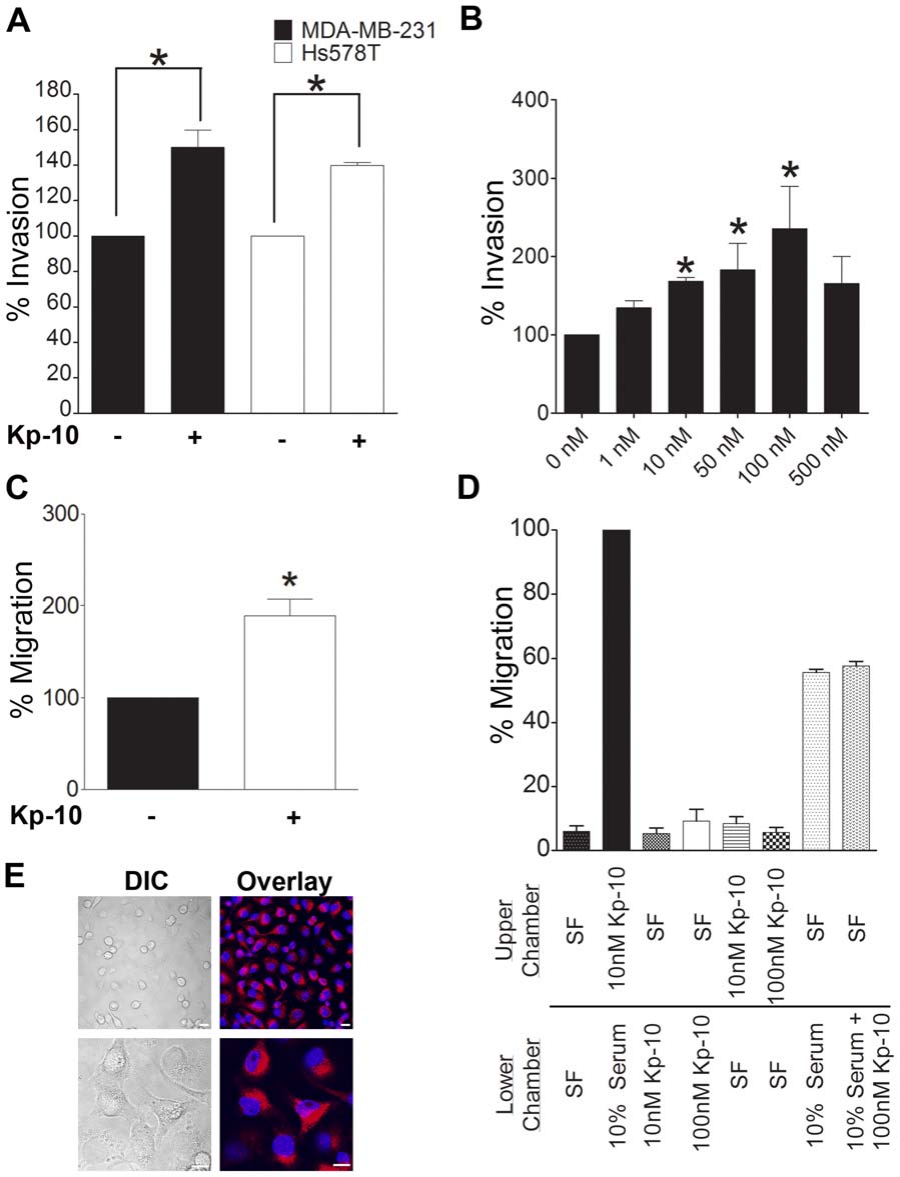
Kp-10 stimulates breast cancer cell invasion. (A) Invasion of MDA-MB-231 and Hs578T cells was measured by Matrigel-coated Transwell chamber assays. Kp-10 treatment (10 nM Kp-10) significantly increased the number of cells invading towards 10% serum. Student's T-test: *, P<0.05 when compared to cells invading towards 10% serum. Data from at least three independent experiments. (B) Kp-10 enhances MDA-MB-231 invasion in a dose-dependent manner. Cell invasion was measured by Matrigel-coated Transwell chamber assays as described above. One-way ANOVA followed by Dunnett's multiple comparison test: *, P<0.05. Data from at least three independent experiments. (C) Kp-10 stimulates breast cancer cell migration of MDA-MB-231 cells as was measured by Transwell chamber assays. One-way ANOVA followed by Dunnett's multiple comparison test: *, P<0.05. Data from at least three independent experiments. (D) Kp-10 does not act as a chemoattractant to stimulate breast cell migration. Migration of MDA-MB-231 cells was measured by Transwell chamber assays. Data from at least three independent experiments. (E) Localization of Kp-10 in MDA-MB-231 cells. Kp-10 immunoreactivity is seen at the cell surface and distributed intracellularly. *Lower panel: cells shown at a higher magnification*. *Scale bar, 10 µm in top panels and 10 µm in bottom panels*. Data from at least three independent experiments.

We first sought to determine whether Kp-10 stimulates invasion of breast cancer cells using standard Transwell chamber Matrigel-invasion assays. Human invasive breast cancer MDA-MB-231 cells or Hs578T cells were seeded in either serum-free media or serum-free media containing Kp-10 and allowed to invade across a Matrigel-coated Transwell membrane for 20 h. We observed that Kp-10 (10 nM) significantly stimulated cell invasion towards 10% serum in both cell-types, compared to the corresponding untreated cells (Fig. 1A). A dose-dependent increase in MDA-MB-231 cell invasion was obtained when treated with various concentrations of Kp-10, with a maximal response obtained using 100 nM Kp-10 (Fig. 1B). All further experiments were done using either 10 nM or 100 nM Kp-10, as reported in other studies [27], [28], [29]. Kp-10 (10 nM) also stimulated MDA-MB-231 cell migration towards 10% serum (Fig. 1C). We found that Kp-10 does not act as a chemoattractant to stimulate breast cell migration (Fig. 1D) or invasion (data not shown). To determine whether or not MDA-MB-231 cells express Kp-10, these cells were immunostained for endogenous Kp-10 using a rabbit anti-Kp-10 antibody (a generous gift from Dr. Alain Caraty, Laboratoire de Physiologie de la Reproduction, France) [21]; we and others have shown that KISS1 and GPR54 are expressed in these cells [13], [28]. We found that Kp-10 positive immunostaining at the cell surface membranes and localized intracellularly in the MDA-MB-231 breast cancer cell line (Fig. 1E).

### Kp-10 promotes breast cancer cell invasion in three-dimensional cultures

**Figure 2:**
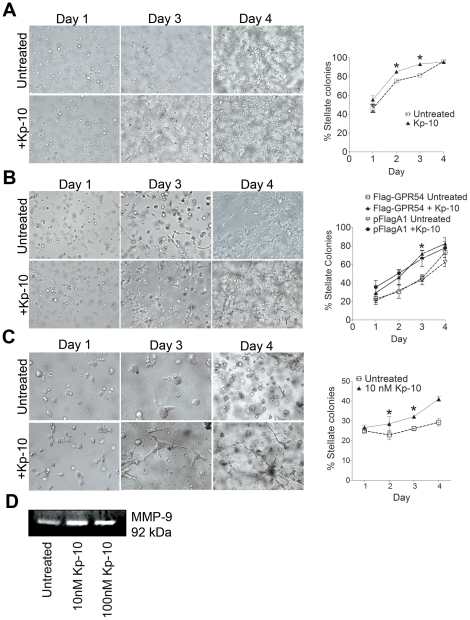
Kp-10 increases breast cancer cell invasive stellate structure formation in 3D cultures by stimulating MMP-9 activity. (A) MDA-MB-231 cells were seeded in a mixture of Matrigel and 10% serum medium. Treatment of cells with 10 nM Kp-10 results in a significant increase in stellate structure formation on days 2 and 3 when compared to untreated cells. Student's t-test: *, *P<0.05* compared to untreated cells invading in Matrigel. Data from at least three independent experiments. (B) Treatment of MDA-MB-231 FLAG-GPR54 cells with 10 nM Kp-10 significantly promotes enhanced stellate structure formation in 3D cultures at day 3 when compared to untreated cells. Student's t-test: *, *P<0.05* compared to untreated stable cell line invading in Matrigel. No statistical difference in degree of stellate structure formation was observed between MDA-MB-231 stably expressing vector, pFlag cells (untreated) and MDA-MB-231 FLAG-GPR54 cells (untreated). Data from at least three independent experiments. (C) Treatment of Hs578T cells with 10 nM Kp-10 significantly enhanced stellate structure formation at days 2 and 3 when compared to untreated cells. Student's t-test: *, *P<0.05* compared to untreated cells invading in Matrigel on their respective days. Data from at least three independent experiments. *Line*, mean percentage of stellate colonies versus total colonies counted that day; *Scale bar, 100 µm in (A) and (B), 40 µm in (C)*. (D) Kp-10 stimulates MMP-9 secretion and activity in breast cancer cells. Representative zymograph from seven independent experiments.

Both normal and malignant breast cells can be cultured in reconstituted extracellular matrix as a three-dimensional (3D) model, mimicking the *in vivo* micro-environment [23]. We have previously demonstrated that MDA-MB-231 cells express endogenous GPR54 [11], [27]. However, since a robust antibody to detect endogenous GPR54 is lacking, we stably expressed FLAG-GPR54 in these cells to easily detect the tagged receptor for biochemical analysis. Treatment of MDA-MB-231 (Fig. 2A), MDA-MB-231 FLAG-GPR54 (Fig. 2B), and Hs578T cells (Fig. 2C) with 10 nM Kp-10 resulted in an increase in the formation of stellate structures, when compared to untreated cells or cells expressing vector control, with a significant increase observed at day 2 and day 3 in MDA-MB-231, day 3 in MDA-MB-231 FLAG-GPR54, and day 2 and day 3 in Hs578T cells. This indicates that Kp-10 treatment of breast cancer cells increases the formation of invasive structures.

### Kp-10 stimulates MMP-9 secretion and activity

In order to determine how Kp-10 promotes breast cancer cell invasion, we employed the use of zymographic analysis of matrix metalloprotease secretion. We found that treatment of MDA-MB-231 cells that express endogenous GPR54 [27] with either 10 or 100 nM of Kp-10 increases the secretion of MMP-9 (Fig. 2D), revealing for the first time that Kp-10 signaling *via* GPR54 in breast cancer cells stimulates the secretion of MMP-9.

### Kp-10 promotes motility of breast cancer cells

**Figure 3:**
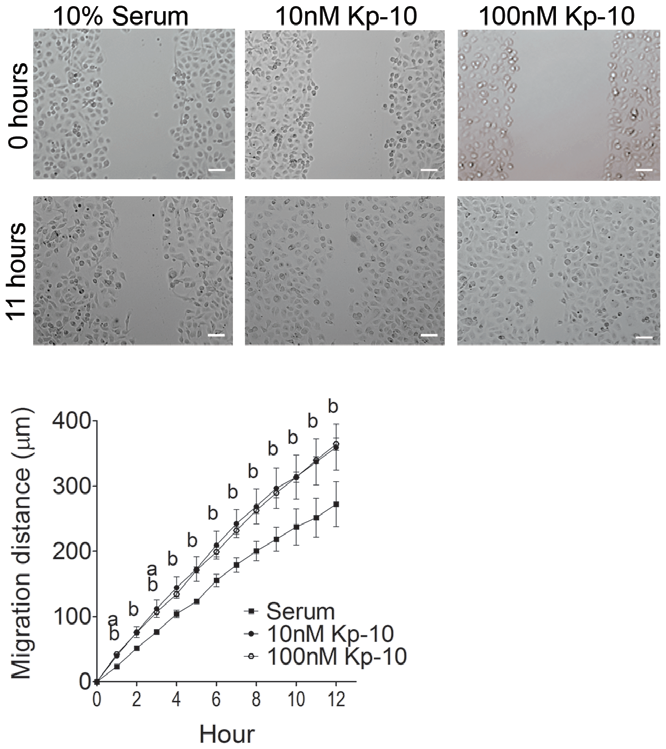
Kp-10 stimulates breast cancer cell motility. Treatment with both 10 nM and 100 nM Kp-10 enhances distance closed by MDA-MB-231 cells in a scratch assay. *Scale bar, 100 µm*. *a*, *P<0.05* for 10 nM Kp-10 when compared to untreated control cells seeded in serum; *b*, *P<0.05* for 100 nM Kp-10 when compared to untreated control cells seeded in serum. Bars represent mean distance travelled in µm ± SEM. Data from five independent experiments.

In order to visualize whether or not Kp-10 stimulates motility of breast cancer cells in real-time, we performed scratch assays as described [30]. We observed that Kp-10 treatments (10 nM or 100 nM) significantly enhance the distance travelled by the MDA-MB-231 cells over time, when compared to cells seeded only in 10% serum (Fig. 3). Taken together, these results suggest that Kp-10 stimulates not only breast cancer cell invasion, but also enhances their motility.

### GPR54 transactivates EGFR in breast cancer cells to regulate cell invasion

**Figure 4:**
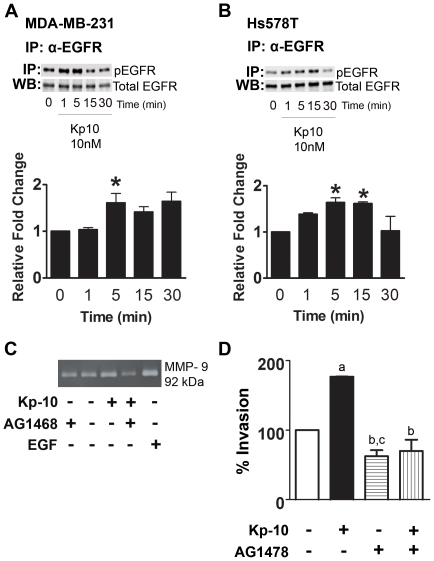
Kp-10 stimulates transactivation of EGFR in breast cancer cells. (A) MDA-MB-231 and (B) Hs578T cells were treated with 10 nM Kp-10 for the indicated time points and then solubilized in lysis buffer. Kp-10 treatment resulted in a significant increase in EGFR phosphorylation (One-way ANOVA followed by Dunnett's multiple comparison test: *, *P<0.05*). Basal levels of phosphorylated EGFR are regarded as 1. Columns represent relative fold change of phosphorylated EGFR as compared to basal level ± SEM. Data from three independent experiments. (C) Kp-10 stimulates MMP-9 secretion and activity in breast cancer cells *via* an EGFR-dependent mechanism. Treatment with AG1468 inhibits Kp-10-dependent MMP-9 secretion. Representative zymograph from three independent experiments. (D) Kp-10 (10 nM) treatment significantly increases MDA-MB-231 FLAG-GPR54 cell invasion. Treatment with AG1478 (500 nM) significantly decreases invasion of MDA-MB-231 FLAG-GPR54 cells *(b, P<0.05*). *a, P<0.05* compared to untreated MDA-MB-231 FLAG-GPR54 cells invading towards media with 10% serum. *b, P<0.05* compared to cells invading towards media with 10% serum. *c, P<0.05* compared to Kp-10-treated MDA-MB-231 FLAG-GPR54 invading towards media with 10% serum. Columns represent the mean percentage of cells invaded as compared to control cells invading towards media with 10% serum ± SEM. Data from three independent experiments.

Since our data revealed that Kp-10 does not act as a chemoattractant to stimulate breast cell migration (Fig. 1D), we investigated whether or not GPR54 may crosstalk with EGFR to regulate breast cancer cell invasion. We initially determined whether or not Kp-10 transactivates this tyrosine kinase receptor. Lysates from Kp-10-treated cells were examined for phosphorylated EGFR by immunoblotting as described [31], [32]. We observed that treatment of MDA-MB-231 cells with 10 nM Kp-10 resulted in an increase in the phosphorylation of EGFR, reaching peak activation at 5 min post-treatment (Fig. 4A). Treatment of Hs578T cells with 10 nM Kp-10 also resulted in increased phosphorylation of EGFR, reaching a peak between 5 and 15 min post-treatment (Fig. 4B) although the Hs578T cells express less EGFR in contrast to MDA-MB-231 cells (Fig. S1A). Both of these invasive breast cancer cell lines exhibit basal EGFR phosphorylation (Fig. 4A, 4B). Thus, our results show for the first time that Kp-10 signaling *via* endogenous GPR54 leads to the transactivation of endogenous EGFR in breast cancer cells.

Since Kp-10 treatment promotes MMP-9 secretion and activity in MDA-MB-231 cells that express endogenous GPR54 [27] (Fig. 2D), we wanted to determine if this was occurring *via* the transactivation of EGFR by GPR54. We found that the activation of MMP-9 was inhibited upon treatment of the cells with AG1468, an EGFR inhibitor, indicating that Kp-10-induced MMP-9 activity was dependent on the activation of EGFR (Fig. 4C). Furthermore, pre-treatment of MDA-MB-231 FLAG-GPR54 cells with AG1478, significantly decreased invasion when compared to untreated cells (Fig. 4D), even in the presence of Kp-10. Thus Kp-10-induced MMP-9 activity required for invasion is dependent on EGFR activity.

### β-arrestin 2 is required for GPR54-mediated invasion

**Figure 5:**
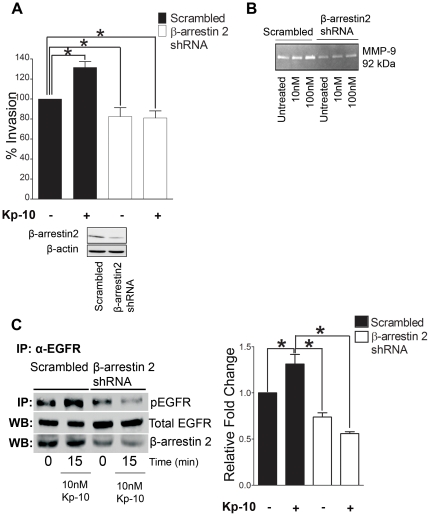
Kp-10-induced breast cancer cell invasion requires β-arrestin 2. (A) MDA-MB-231 cells stably expressing scrambled shRNA controls and β-arrestin 2 shRNA were plated in serum-free medium or serum-free medium containing 10 nM Kp-10 and allowed to invade for 20 h towards media with 10% serum. Kp-10 treatment (10 nM) significantly increased invasion of cells expressing scrambled control towards 10% serum (*, *P<0.05*). Depletion of β-arrestin 2 significantly inhibits invasion towards media with 10% serum when compared to Kp-10-treated control cells (**, *P<0.05*). Columns represent mean percentage of cells invaded as compared to control cells invaded towards media with 10% serum ± SEM. Data from three independent experiments. (B) Depletion of β-arrestin 2 blocks MMP-9 secretion and activity in breast cancer cells. MDA-MB-231 cells stably expressing scrambled control shRNA or β-arrestin 2 shRNA were treated for 48 h in Kp-10 in serum-free media. Overlaid media was then collected and subjected to gelatin zymography. Data from five independent experiments. (C) Depletion of β-arrestin 2 inhibits Kp-10–mediated EGFR phosphorylation in MDA-MB-231 cells. Cells were serum-starved for 24 h, treated with 10 nM Kp-10 for the indicated time points. Kp-10 treatment results in a significant increase in EGFR phosphorylation in the scrambled controls, in contrast to β-arrestin 2 knockdown cells (One-way ANOVA followed by Dunnett's multiple comparison test: *, *P<0.05*). Basal levels of phosphorylated EGFR are regarded as 1. Columns represent relative fold change of phosphorylated EGFR as compared to basal level ± SEM. Data from three independent experiments.

We have recently demonstrated that the GPCR adaptor protein, β-arrestin, regulates MDA-MB-231 cell invasiveness [23]. In addition, we found that GPR54, which is endogenously expressed in MDA-MB-231 cells, was unable to stimulate ERK1/2 activity, upon depletion of β-arrestin 2 [27]. This suggests that β-arrestin 2 is required for GPR54 signaling to ERK1/2. To define a molecular mechanism by which Kp-10 induces breast cancer cell invasiveness, we investigated the effect of depleting β-arrestin 2 on this process. Knockdown of β-arrestin 2 in MDA-MB-231 cells significantly reduced basal invasion in the absence of Kp-10, as well as Kp-10-stimulated invasion, compared with the scrambled control shRNA (Fig. 5A). Furthermore, we found that depletion of β-arrestin 2 reduced MMP-9 secretion and activity in cells, in the presence or absence of Kp-10, in contrast to cells expressing the scrambled control shRNA (Fig. 5B). Kp-10–mediated EGFR phosphorylation was also inhibited in β-arrestin 2 knockdown cells, as compared to the scrambled controls, implicating a role for β-arrestin 2 in mediating Kp-10-induced EGFR transactivation (Fig. 5C). Knockdown of β-arrestin 2 did not affect cell viability as determined by 3-(4,5-dimethylthiazol-2-yl)-2,5-diphenyltetrazolium bromide (MTT) assays (Fig. S1B). These results reveal that β-arrestin 2 regulates basal and Kp-10-dependent invasion, by regulating MMP-9 secretion and activity.

Although the details of the mechanism by which endogenous β-arrestins regulate MMP activity is currently unknown, β-arrestins can scaffold Src, shown previously to promote invasion *via* MMP-9 secretion in breast cancer cells [33], [34]. However, we found that knockdown of β-arrestin 2 expression had no effect on Src activation in MDA-MB-231 cells (Fig. S2), implicating that other pathways might be involved.

### Kp-10 or EGF stimulates the internalization of GPR54 in breast cancer cells

**Figure 6:**
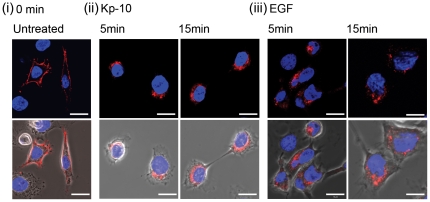
Kp-10 and EGF induce internalization of GPR54 and EGFR in breast cancer cells. MDA-MB-231 cells stably expressing FLAG-GPR54 were grown on glass coverslips and incubated in the absence (0 min) or presence of Kp-10 (10 nM) or EGF (10 ng/mL). Cells were then subjected to immunofluorescence staining using a monoclonal FLAG antibody (red) and nuclei staining with 0.1% Hoechst 33258 dye (blue). Upon treatment of cells with 10 nM Kp-10 (ii) or 10 ng/mL EGF (iii), FLAG-GPR54 internalized and was localized at peri-nuclear regions. Representative confocal micrographs from five independent experiments are shown. *Scale bar, 10 µm*.

Our data so far indicates that GPR54 transactivates EGFR to regulate breast cancer cell invasion. To better understand the nature of the cross-talk between these two receptors, we sought to determine whether or not Kp-10 or EGF can stimulate GPR54 endocytosis in MDA-MB-231 FLAG-GPR54-expressing cells. FLAG-GPR54 was localized at the cell surface in unstimulated cells (Fig. 6-i). Treatment with 10 nM Kp-10 or 10 ng/mL EGF (Fig. 6-ii and -iii, respectively) resulted in FLAG-GPR54 receptor internalization within 5 or 15 min of stimulation to a peri-nuclear region. This demonstrated that EGF, like Kp-10, induces the internalization of GPR54 in breast cancer cells.

### GPR54 associates with EGFR

**Figure 7:**
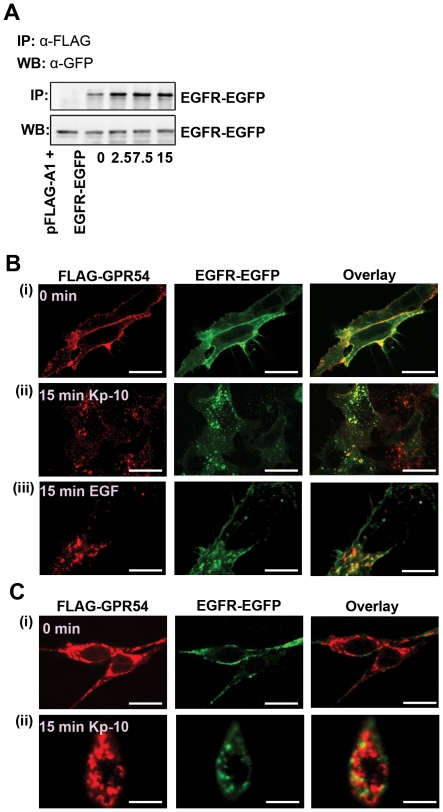
GPR54 associates with EGFR. (A) GPR54 co-immunoprecipitates EGFR in HEK 293 cells expressing FLAG-GPR54 and EGFR-GFP in the presence or absence of Kp-10 (100 nM). Representative blot of three independent experiments shown. (B) HEK 293 cells expressing FLAG-GPR54 and EGFR-GFP were incubated in the absence (0 min, i) or presence of Kp-10 (10 nM, ii) or EGF (10 ng/mL, iii) for 15 min. After 15 minutes of Kp-10 stimulation (ii), FLAG-GPR54 and EGFR-GFP partially co-localized in endocytotic vesicles (*yellow*). Similar results were obtained upon treatment of cells with 10 ng/mL EGF for 15 minutes (iii). Representative blot of three independent experiments shown. *Scale bar, 10 µm*. C) MDA-MB-231 cells stably expressing FLAG-GPR54 and EGFR-EGFP were incubated in the absence (0 min, i) or presence of Kp-10 (10 nM) for 15 min (ii). Representative confocal images of five experiments shown here. *Scale bar, 10 µm*.

We also determined whether or not GPR54 is able to interact with EGFR using co-immunoprecipitation studies. We found that FLAG-GPR54 interacts with EGFR in the absence of Kp-10 in HEK 293 cells and that addition of Kp-10 (100 nM) increased the amount of interaction of GPR54 with EGFR (Fig. 7A). To determine whether or not GPR54 and EGFR are co-localized to the same endocytotic vesicles upon internalization, we co-expressed FLAG-GPR54 and EGFR-GFP in cells. In HEK 293 cells, both receptors were localized at the cell membranes (Fig. 7B-i). Treatment of cells with either Kp-10 (Fig. 7B-ii) or EGF (Fig. 7B-iii) resulted in partial co-localization of FLAG-GPR54 and EGFR-GFP in endocytotic vesicles. In unstimulated MDA-MB-231 cells, FLAG-GPR54 and EGFR-GFP were primarily localized to the cell membrane (Fig. 7C-i). Treatment with 10 nM Kp-10 resulted in internalization of both FLAG-GPR54 and EGFR-EGFP, with partial co-localization of the two receptors upon Kp-10 treatment (Fig. 7C-ii). Similar observations were made upon treatment of cells with EGF (data not shown).

### GPR54 interacts directly with EGFR

**Figure 8:**
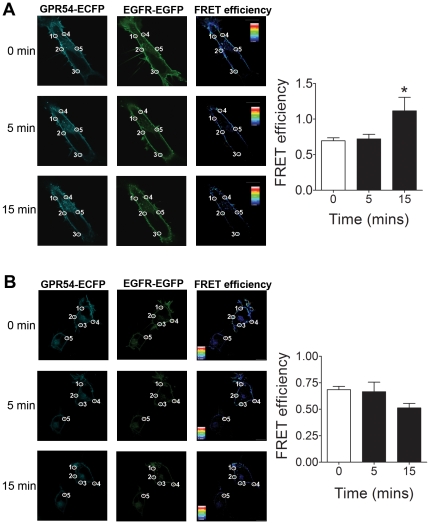
GPR54 interacts directly with EGFR. PFRET analysis of HEK 293 cells expressing GPR54-ECFP and EGFR-EGFP at the plasma membrane before and after stimulation with (A) 100 nM Kp-10 and (B) 10 ng/ml EGF. Representative confocal micrographs are shown. *Scale bar, 10 µm*. The images show the location of the 5 ROIs at the plasma membrane where FRET was analyzed. The left column show the localization of GPR54-ECFP, the middle column shows the localization of EGFR-EGFP, and the right column shows the results for FRET efficiency after performing the PFRET analysis using the sensitized emission method (see colour scale for reference). Columns represent mean PFRET values ± SEM. Data from five independent experiments.

To further confirm the interaction between GPR54 and EGFR, and to study the nature of this interaction, we performed PFRET on HEK 293 cells co-expressing GPR54-ECFP and EGFR-EGFP; in all cases PFRET analysis was performed at the plasma membrane. Our results confirmed our co-immunoprecipitation experiments, as we observed a high FRET efficiency in the plasma membrane of unstimulated cells expressing both receptors (Fig. 8). These results indicate that GPR54 interacts directly with EGFR at the plasma membrane even in the absence of agonist. Following stimulation with Kp-10 (100 nM) or EGF (10 ng/ml), both receptors remained associated with each other at the plasma membrane; however, only stimulation with Kp-10 significantly changed the average FRET efficiency values relative to that of unstimulated cells upon 15 min of stimulation (Fig. 8A, B). Similar results were obtained upon stimulation with 10 nM Kp-10 (data not shown). To account for the reduction in fluorescence caused by photobleaching of the fluorophores after repeated imaging of the same set of cells, we performed control experiments with unstimulated cells and imaged them at 5 and 15 min. The average FRET efficiency values obtained from these controls were used to normalize the data obtained in cells stimulated with Kp-10 and EGF (Fig. 8A, B). We did not observe a significant amount of FRET signal inside the cytoplasm of non-treated or agonist-stimulated cells. Overall, these data indicate that GPR54 interacts directly with EGFR in the presence or absence of agonist.

## Discussion

Although recent studies indicate that Kp signaling may correlate positively with breast tumor progression and metastatic potential [10], [11], it is unknown whether Kp stimulate breast cancer cell invasiveness. Our data suggest that Kp-10/GPR54 signaling is pro-migratory and pro-invasive in human breast cancer cells. We show for the first time that Kp-10 stimulates breast cancer cell invasion concomitant with MMP-9 secretion and activity, and have implicated β-arrestin 2 in this process. We have also discovered that GPR54 directly complexes with EGFR, and that stimulation of breast cancer cells with either Kp-10 or EGF regulates the endocytosis of both GPR54 and EGFR.

Significant progress has been made in understanding the complexity of GPCR tyrosine kinase signaling over the last decade. Once seen as isolated receptors connecting extracellular signals to the activation of G proteins, GPCRs are now regarded as complex receptors capable of initiating a vast array of signaling pathways, including G protein-dependent and -independent signaling, binding various scaffolding molecules, and interacting both directly and indirectly with other receptor families. Several GPCRs have been shown to transactivate EGFR and this plays important roles in not only normal physiological functions, but also in pathophysiology [17], [49]–[53].

Elevated expression of EGFR is associated with highly aggressive and metastatic cancers, including cancer of the breast [35]. As a member of the ErbB family of receptor tyrosine kinases, EGFR has been characterized as a proto-oncogene. EGFR and other ErB receptors may be upregulated or mutated in cancer and this results in changes in downstream signaling partners which can contribute to resistance mechanisms employed by cancer cells. This ability of EGFR, to function despite treatment with tyrosine kinase inhibitors and targeting antibodies, poses a major problem for cancer therapy [35]. Thus, an understanding of EGFR signaling cascades is vital in the production of specific therapies targeting metastasis.

Our findings reveal that unlike their role in other cancer cell types, Kp-10 positively regulates breast cancer cell motility and invasion. Kp-10 does not act as a chemoattractant but potentiates invasion towards 10% serum. MDA-MB-231 and Hs578T cells are negative for the expression of estrogen receptors; estrogen receptor levels are used as a prognostic marker of breast tumors [36]. We investigated the effects of Kp-10 on cell migration of human T47D cells which also express KISS1 [10], but are positive for estrogen and progesterone receptors. We found that Kp-10 (10 or 100 nM) did not stimulate T47D cell migration (Fig. S3A), suggesting that Kp-10 may influence metastatic potential of the more aggressive breast cancer cells that are deficient of hormone receptors. Similarly, we found that Kp-10 (10 or 100 nM) did not stimulate migration of the human malignant melanoma cells C8161 (Fig. S3B), which have been previously used by Welch's group to study the anti-metastatic effects of Kp [1].

We further show that Kp-10-mediated activation of GPR54 enhances the invasive abilities of MDA-MB-231, MDA-MB-231 FLAG-GPR54, and Hs578T cells in 3D invasion assays. Kp-10 did not enhance invasion of breast cancer cells when EGFR was inhibited through the use of tyrosine kinase inhibitor AG1478, suggesting EGFR involvement in Kp-10-stimulated invasion. Previous reports have shown that MDA-MB-231 invasion towards EGF to be blocked by AG1478 [37]. We found that Kp-10 transactivates EGFR robustly in MDA-MB-231 cells and in Hs578T cells that are less metastatic [38] and express significantly less EGFR, as we and others have shown [39].

Our data reveals that Kp-10 can stimulate invasiveness concomitant with the production of MMP-9, and that this is dependent on EGFR activation. To date, this is the first demonstration that Kp stimulate MMPs. Conversely, it has been reported that in HT-1080 human fibrosarcoma cells where Kp signaling is anti-invasive, expression of *KISS1* results in the negative regulation of MMP-9 production [9]. We found that Kp-10-induced EGFR transactivation and invasion occur *via* a β-arrestin 2-dependent mechanism. Furthermore, transactivation of EGFR by GPR54 is required for the secretion and activation of MMP-9 in addition to β-arrestin 2. We have recently shown that the β-arrestins play important roles in regulating breast cancer cell invasion towards the bioactive lipid lysophosphatidic acid *via* Ral GTPases [23], [40]. Several other studies have shown that β-arrestins participate in tumor-related signaling pathways [41], [42], [43], [44], [45]. Recent work has shown that transgenic mice over-expressing β-arrestin 1 resulted in rapid xenograft tumor progression due to enhanced tumor angiogenesis, mediated by an increase in MMP-9 activity [45]. Therefore, β-arrestins may function as critical regulators of tumor invasion and these studies are currently under investigation.

Kp-10 can stimulate cytoskeletal changes required for cell motility *via* Rho GTPases [3]. A role for RhoA has been shown in regulating cell migration in highly aggressive cancer cells such as MDA-MB-231 and MDA-MB-435 [46]. Constitutively active Rho mutant V14 was shown in MDA-MB-231 cells to not only increase basal migration, but also to enhance basal EGFR activation [47]. Activation of RhoA and dephosphorylation of FAK, processes responsible for EGF-induced cell retractile morphological changes, occurs upon EGF treatment in EGFR-overexpressing cells [48]. Thus, Kp treatment of MDA-MB-231 cells may promote migration by stimulating RhoA activation and FAK dephosphorylation *via* EGFR.

We show for the first time that GPR54 directly interacts with EGFR by PFRET using the sensitized emission method. Our results show that the two receptors interact with each other in unstimulated cells, and this association occurs mainly at the plasma membrane. Following agonist stimulation, a fraction of receptors continue to associate at the plasma membrane, although we did observe receptor internalization by confocal studies. The lack of a substantial amount of FRET signal inside cells following agonist stimulation could be due to the fact that the interaction between the two receptors is no longer direct, once the receptors have internalized. It is important to note that FRET methods based on fluorescence intensity, such as the sensitized emission method that we used in this study, cannot distinguish between a change in FRET efficiency and a change in FRET population [49]. Therefore, a reduction in the number of receptor molecules present at the plasma membrane due to agonist-stimulated internalization would not affect the average FRET values. Furthermore, the absence of FRET does not provide proof for the absence of an interaction, since there are a number of factors that can affect the results, such as a change in the physical structure of the receptor molecules that would separate the donor and acceptor moieties or movement of the receptor molecules as they internalize out of the focal plane [50].

To date, a limited number of GPCRs have been shown to interact (directly or indirectly) with EGFR. Recently, it was discovered that the vasopressin 1A receptor, a G_q_–coupled receptor also interacts with EGFR by co-immunoprecipitation studies, although how this interaction affects signaling of either receptor is unknown [51]. The β1-adrenergic receptor has been shown to interact with EGFR by co-immunoprecipitation and FRET analysis under basal conditions [52]. Treatment of cells with the β1- adrenergic receptor agonist, isoproterenol, did not affect FRET efficiency when compared to untreated cells [52]. We also found that EGF treatment promoted the internalization of GPR54 in breast cancer cells and that GPR54 and EGFR partially co-localized upon treatment of Kp-10 or EGF. Similar observations have been reported with β2-adrenergic receptor which co-localized with EGFR in response to isoproterenol stimulation [53]. Thus, although many GPCRs can transactivate EGFR [16], [54], [55], the association with EGFR appears to be unique to a subset of GPCRs. For example, activation of the angiotensin receptor (AT_1A_R) can also transactivate EGFR, however this receptor does not complex with EGFR [52].

Targeted therapies against EGFR in cancer treatment have been disappointing due to the robust nature of EGFR signalling. Our work provides the first evidence that GPR54 signaling positively regulates breast cancer cell invasiveness, *via* EGFR crosstalk that involves MMPs and β-arrestin 2. Currently, ongoing studies are looking to identify the binding domains between both receptors, and to further examine the nature of the crosstalk. Elucidation of novel signaling pathways may uncover new drug targets.

## Supporting Information

Figure S1 **EGFR expression in breast cancer cells and cell viability assays.** (A) Comparison of total EGFR expression in Hs678T, MDA-MB-231 and FLAG-GPR54 MDA-MD-231 by Western blot analysis. Data from three independent experiments. (B) Knockdown of β-arrestin 2 does not affect cell viability as determined by 3-(4,5-dimethylthiazol-2-yl)-2,5-diphenyltetrazolium bromide (MTT) assays. Data from three independent experiments.
(TIF)

Figure S2 **The effect of β-arrestin 2 knockdown on Src phosphorylation.** Depletion of β-arrestin 2 has no effect on the change in Src phosphorylation (phospho-Src family Tyr416, 1∶1000, Cell Signaling Technology) levels in MDA-MB-231 β-arrestin 2 knockdown cells when compared to the MDA-MB-231 scrambled. MDA-MB-231 scrambled and MDA-MB-231 β-arrestin 2 knockdown were serum-starved for 24 h, treated with 10 nM Kp-10 for the indicated time points. Densitometric analysis of western blots revealed that following Kp-10 treatment there was no change in Src phosphorylation in the scrambled controls or in the MDA-MB-231 β-arrestin 2 knockdown cells. Basal levels of phosphorylated Src are regarded as 1. Columns represent relative fold change of phosphorylated EGFR as compared to basal level ± SEM. Data from three independent experiments.
(TIF)

Figure S3 **The effect of Kp-10 on T47D and C8161 cell migration.** Kp-10 does not stimulate migration of (A) T47D breast cancer cell or (B) C8161 invasive melanoma cells as measured by Transwell chamber assays. Data from six independent experiments.
(TIF)
