# Supplementary figures and images for: GPR54 (KISS1R) Transactivates EGFR to Promote Breast Cancer Cell Invasiveness
Source: PLoS One. 2011 Jun 28;6(6):e21599. doi: 10.1371/journal.pone.0021599 (PMC3125256; doi:10.1371/journal.pone.0021599)

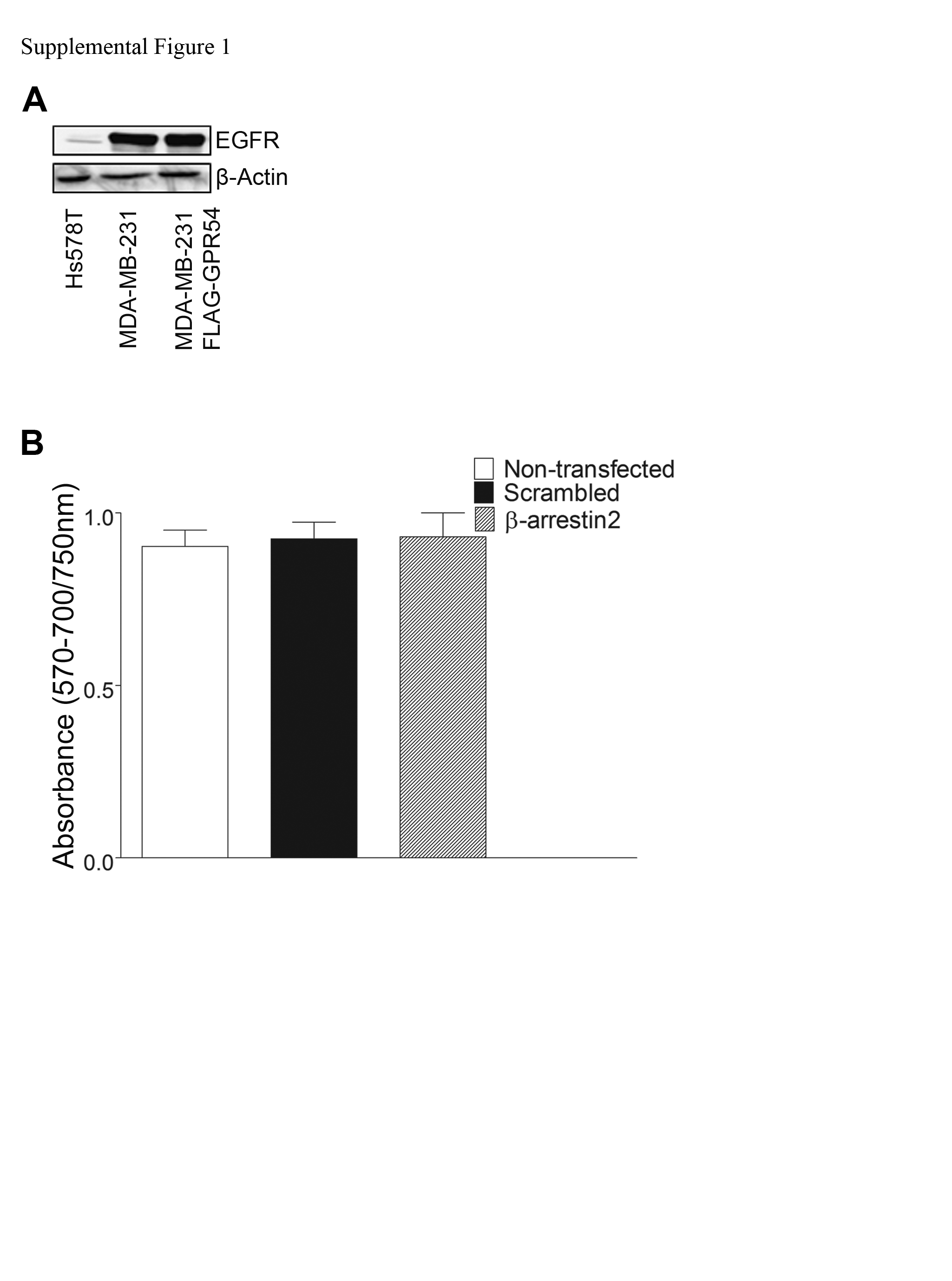

Supplement: Figure S1 — EGFR expression in breast cancer cells and cell viability assays. (A) Comparison of total EGFR expression in Hs678T, MDA-MB-231 and FLAG-GPR54 MDA-MD-231 by Western blot analysis. Data from three independent experiments. (B) Knockdown of β-arrestin 2 does not affect cell viability as determined by 3-(4,5-dimethylthiazol-2-yl)-2,5-diphenyltetrazolium bromide (MTT) assays. Data from three independent experiments. (TIF) [file pone.0021599.s001.tif]

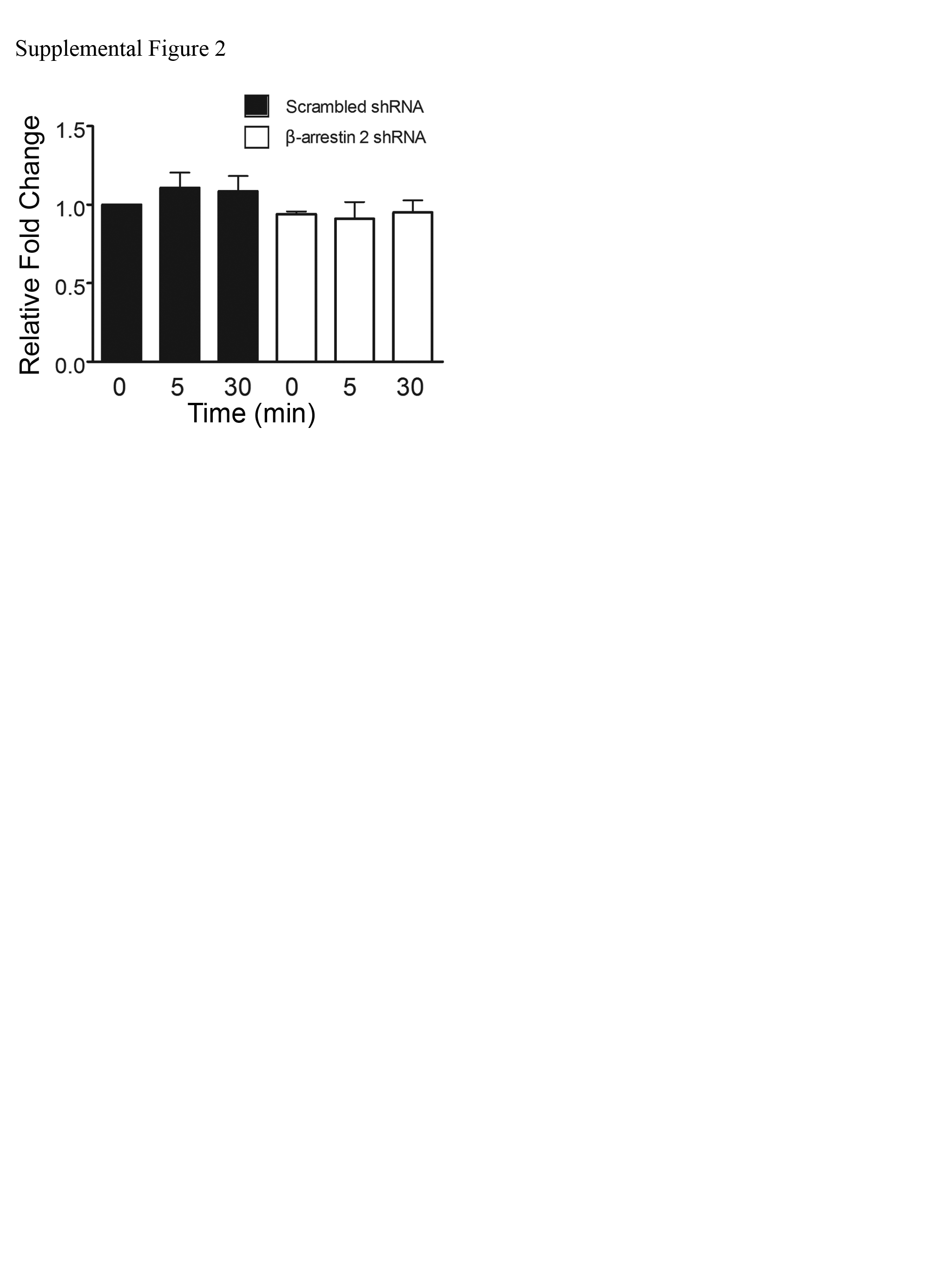

Supplement: Figure S2 — The effect of β-arrestin 2 knockdown on Src phosphorylation. Depletion of β-arrestin 2 has no effect on the change in Src phosphorylation (phospho-Src family Tyr416, 1∶1000, Cell Signaling Technology) levels in MDA-MB-231 β-arrestin 2 knockdown cells when compared to the MDA-MB-231 scrambled. MDA-MB-231 scrambled and MDA-MB-231 β-arrestin 2 knockdown were serum-starved for 24 h, treated with 10 nM Kp-10 for the indicated time points. Densitometric analysis of western blots revealed that following Kp-10 treatment there was no change in Src phosphorylation in the scrambled controls or in the MDA-MB-231 β-arrestin 2 knockdown cells. Basal levels of phosphorylated Src are regarded as 1. Columns represent relative fold change of phosphorylated EGFR as compared to basal level ± SEM. Data from three independent experiments. (TIF) [file pone.0021599.s002.tif]

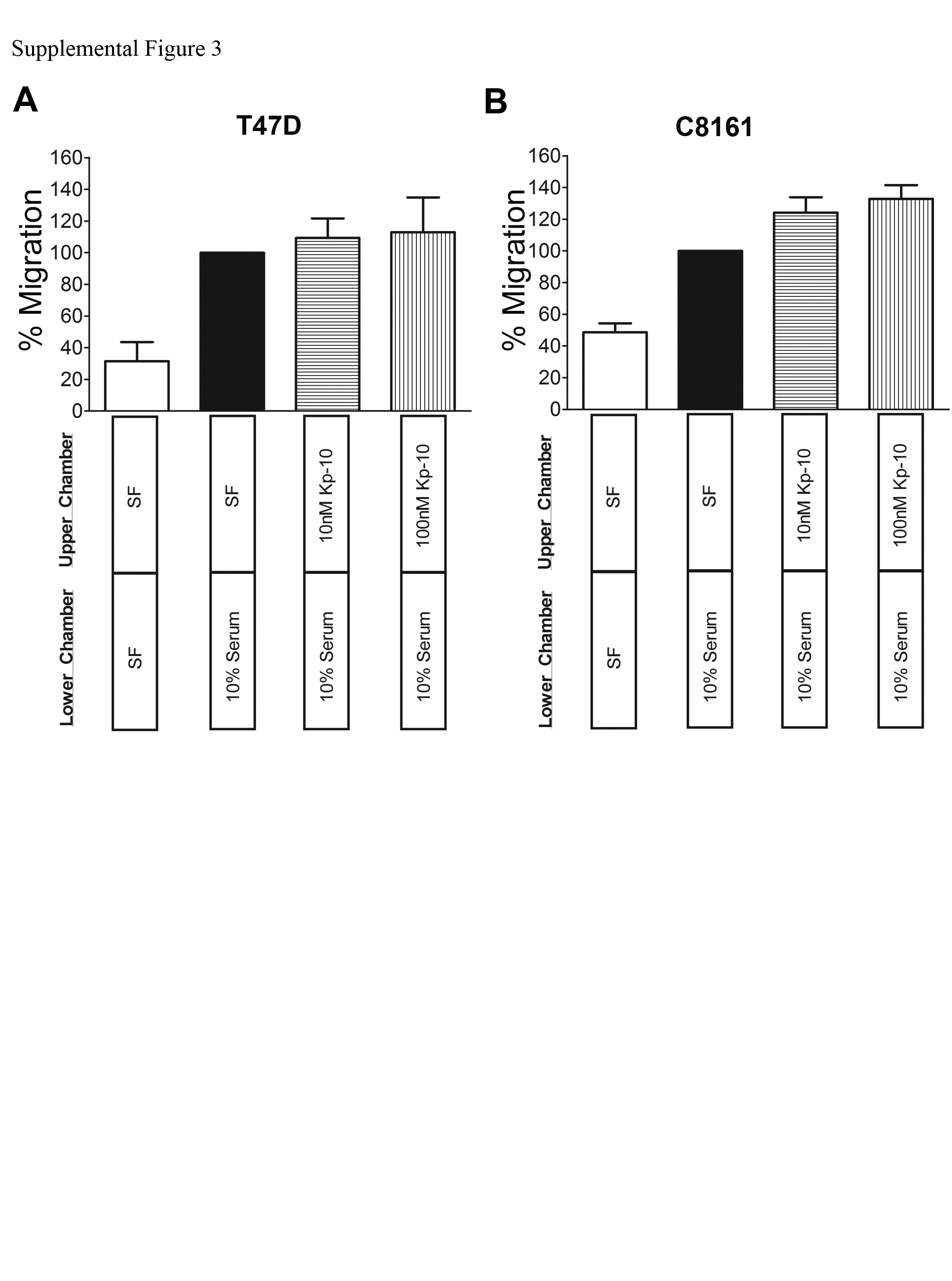

Supplement: Figure S3 — The effect of Kp-10 on T47D and C8161 cell migration. Kp-10 does not stimulate migration of (A) T47D breast cancer cell or (B) C8161 invasive melanoma cells as measured by Transwell chamber assays. Data from six independent experiments. (TIF) [file pone.0021599.s003.tif]
